# Sustained activation of detoxification pathways promotes liver carcinogenesis in response to chronic bile acid-mediated damage

**DOI:** 10.1371/journal.pgen.1007380

**Published:** 2018-05-07

**Authors:** Agnese Collino, Alberto Termanini, Paola Nicoli, Giuseppe Diaferia, Sara Polletti, Camilla Recordati, Vittoria Castiglioni, Donatella Caruso, Nico Mitro, Gioacchino Natoli, Serena Ghisletti

**Affiliations:** 1 Department of Experimental Oncology, European Institute of Oncology (IEO), Milan, Italy; 2 Humanitas Clinical and Research Center, Rozzano, Milan, Italy; 3 Humanitas University, Pieve Emanuele, Milan, Italy; 4 Mouse & Animal Pathology Laboratory, Fondazione Filarete, Milan, Italy; 5 DiSFeB, Dipartimento di Scienze Farmacologiche e Biomolecolari, Università degli Studi di Milano, Milano, Italy; Sanford-Burnham Medical Research Institute at Lake Nona, UNITED STATES

## Abstract

Chronic inflammation promotes oncogenic transformation and tumor progression. Many inflammatory agents also generate a toxic microenvironment, implying that adaptive mechanisms must be deployed for cells to survive and undergo transformation in such unfavorable contexts. A paradigmatic case is represented by cancers occurring in pediatric patients with genetic defects of hepatocyte phosphatidylcholine transporters and in the corresponding mouse model (*Mdr2*^-/-^ mice), in which impaired bile salt emulsification leads to chronic hepatocyte damage and inflammation, eventually resulting in oncogenic transformation. By combining genomics and metabolomics, we found that the transition from inflammation to cancer in *Mdr2*^-/-^ mice was linked to the sustained transcriptional activation of metabolic detoxification systems and transporters by the Constitutive Androstane Receptor (CAR), a hepatocyte-specific nuclear receptor. Activation of CAR-dependent gene expression programs coincided with reduced content of toxic bile acids in cancer nodules relative to inflamed livers. Treatment of *Mdr2*^-/-^ mice with a CAR inhibitor blocked cancer progression and caused a partial regression of existing tumors. These results indicate that the acquisition of resistance to endo- or xeno-biotic toxicity is critical for cancers that develop in toxic microenvironments.

## Author summary

Chronic inflammation associated to the sustained exposure to toxic chemicals can lead to cancer, but how transforming cells acquire the ability to oppose chemo-toxicity and eventually thrive is unclear. In this study, we set out to profile the molecular changes occurring in a mouse model of liver disease caused by defective emulsification of bile acids, which leads to membrane damage, cell death, chronic inflammation and eventually cancer. We found that hepatocytes acquire early in tumorigenesis the ability to efficiently cope with the accumulation of toxic bile acids by increasing the expression of detoxification pathway components via both genetic and epigenetic mechanisms.

## Introduction

The microenvironment of chronically inflamed tissues is a source of multiple mediators that trigger and sustain cellular transformation and tumorigenesis [1–4]. Within the broad and heterogeneous group of inflammation-associated cancers, a distinct class is represented by those tumors that develop within a microenvironment containing high concentrations of toxic substances causing chronic cellular damage and compensatory tissue regeneration.

A straightforward logical assumption is that for cells to emerge, thrive and eventually develop cancers in such contexts, they must acquire early in tumorigenesis the ability either to efficiently cope with the damage exerted by toxic agents or to promote their detoxification. To directly test this hypothesis, we used a well-characterized model of liver cancer, in which the absence of ABCB4, a transporter for phosphatidylcholine expressed selectively in hepatocytes and encoded by the *Mdr2* gene, results in defective emulsification of bile acids and their precipitation on the bile canalicular surface of hepatocytes, thus leading to membrane damage, cell death and chronic inflammation. In the absence of any exogenous mutagen, *Mdr2*^-/-^ animals develop liver cancers with 100% penetrance at 12–15 months of age [5–9]. These cancers are etiologically and genetically similar to those occurring in pediatric patients with type 2 Progressive Familial Intrahepatic Cholestasis (PFIC), in which mutations in the same family of hepatocyte transporters results in liver cancer by the age of five [10,11].

To understand the molecular bases of cancer development in this specific context, in which cellular toxicity is caused by chronic exposure to non-neutralized endogenous compounds (namely, non-micellar hydrophobic bile acids), we performed gene expression, epigenomic and metabolomic profiling in hepatocytes to identify changes in gene expression programs and regulatory networks associated first with inflammation and then with cancer development. We found that while chronic liver inflammation was associated with the induction of a stress-response characterized by the induction of metalloproteinases and collagen genes among the others, Hepatocellular Carcinoma (HCC) development was characterized by the downregulation of these inflammatory programs and instead a robust transcriptional activation of genes encoding enzymes involved in the two phases of metabolic transformations and detoxification, namely Phase I (oxidation, reduction and hydrolysis) and Phase II transformations (conjugation, *e*.*g*. to glutathione), as well as efflux transporters involved in the extrusion of transformed metabolites from cells. The induction of such response was associated with reduced content of toxic bile acids in cancer nodules relative to inflamed livers. Computational mining of the genomic data indicated that this gene expression program was driven by the increased expression and activation of the hepatocyte-specific Constitutive Androstane Receptor (CAR, encoded by the *Nr1i3* gene), a transcription factor of the nuclear receptor superfamily known to control xenobiotic detoxification genes. Consistent with these data, CAR inhibition with a specific antagonistic ligand reduced tumor burden and resulted in the regression of cancer nodules.

Altogether, our data suggest that by mounting an appropriate detoxification response, hepatocytes became able to cope with the accumulation of toxic bile acids during liver inflammation, thus acquiring the capacity to thrive and undergo neoplastic transformation in an otherwise toxic environment.

## Results

### Hepatocyte gene expression programs in *Mdr2^-/-^* mice

We first performed RNA sequencing (RNA-seq) to analyze the changes in transcriptome of hepatocytes during *Mdr2^-/-^* liver disease progression. *Mdr2* is expressed selectively in hepatocytes, thus justifying the use of a full knockout for these experiments. Hepatocytes represent more than 75% of the cell populations of a normal liver. However, immunohistochemistry (IHC) staining of liver sections showed a massive infiltration of IBA-1 positive macrophage cells in inflamed livers of 8-months old mice and even more so in HCC nodules (15 to 17 months old mice) (**S1A** and **S1B Fig**). Moreover, attempts to isolate pure hepatocytes after collagenase perfusion via the portal vein of *Mdr2^-/-^* mice were not successful because of the extensive co-purification of macrophages. Therefore, to obtain hepatocyte-enriched liver samples we treated mice with liposomes loaded with clodronate to deplete liver macrophages [12]. The 48 hours clodronate treatment resulted in a significant macrophage depletion from both inflamed livers and cancer nodules of *Mdr2^-/-^* mice, as shown in **S1A** and **S1B Fig**. An RNA-seq analysis carried out in livers of untreated and clodronate treated mice revealed that genes differentially expressed by clodronate treatment were significantly enriched for ontology terms associated to macrophage and lymphocyte function (**S1C Fig**, **S1 Table**). In addition to macrophage depletion, clodronate treatment reduced B and T lymphocytes content in the normal liver but it had no significant effect on the inflamed and neoplastic livers of *Mdr2^-/-^* mice (**S2 Fig**).

**Fig 1:**
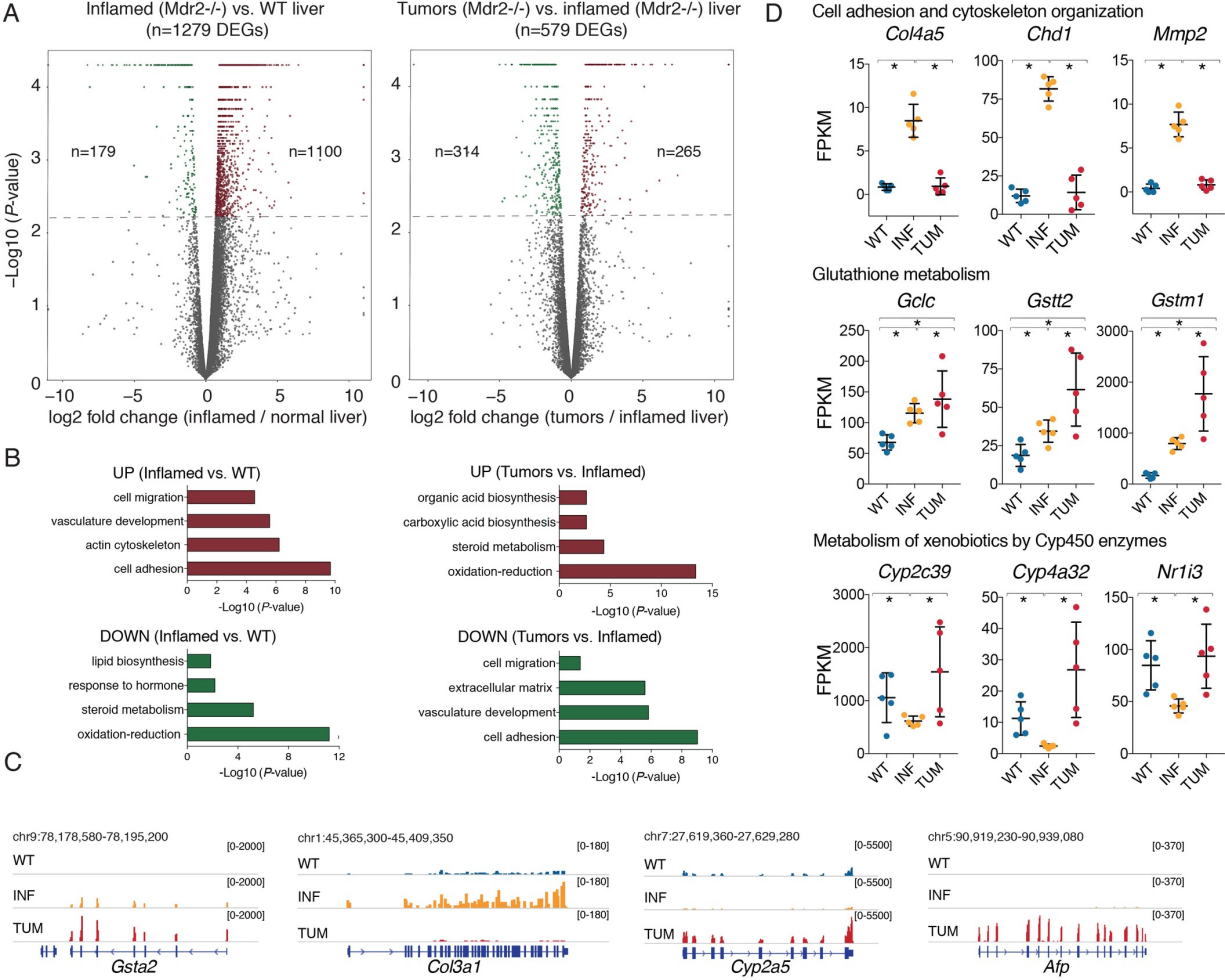
Transcriptional alterations accompanying *Mdr2^-/-^* liver disease progression. **(A)** Volcano plots reporting differentially expressed genes (up-regulated brown, down-regulated green) identified by comparing WT and inflamed *Mdr2^-/-^* livers (left panel) and *Mdr2^-/-^* inflamed livers and tumors (right panel). The y-axis shows the -Log10 of the P-values determined by Cuffdiff analysis. **(B)** Gene ontology analyses on the sets of differentially expressed genes (up- and down-regulated) in each comparison. **(C)** Representative RNA-seq snapshots of differentially expressed genes. **(D)** Representative genes belonging to cell adhesion and ECM/cytoskeleton organization (*Col4a5*, *Chd1*, *Mmp2*), to glutathione metabolism (*Gclc*, *Gstt2*, *Gstm1*,) and to metabolism of xenobiotics by CYP450 enzymes (*Cyp2c39*, *Cyp4a32*, *Nr1i3*). Differences were assessed using two-sided Mann-Whitney test (p < 0.05).

Therefore, we used this approach to generate RNA-seq data sets in clodronate-treated and macrophage-depleted livers, including: *i*) inflamed livers of 8 months old *Mdr2^-/-^* mice; *ii*) isolated nodules from 15-to-17-months old *Mdr2^-/-^* mice, and *iii*) age matched FVB/NJ wild type mice. All samples were sequenced to an average depth of ~30 million paired-end reads, using five animals per experimental group (**S3 Fig**). Of the 1279 differentially expressed genes (DEGs) identified in the comparison of inflamed *vs*. wild type livers (fold change ≥ ∣2∣, q-value ≤ 0.05, FPKM ≥ 2 in at least one time point), 1100 (86%) were upregulated in the inflamed livers, consistent with a widespread increase in the expression of inflammatory genes (**Fig 1A**, left panel). In the comparison between inflamed livers and HCC nodules, gene expression changes were of comparatively lower amplitude (579 DEGs) and they occurred similarly in both directions: 265 genes (45.8%) were up regulated and 314 (54.2%) were down-regulated (**Fig 1A**, right panel; the complete list of genes is reported in **S2 Table** and a Q-PCR validation of selected DEGs is reported in **S4 Fig**).

A gene ontology (GO) analysis of the genes differentially expressed in inflamed *vs*. WT livers and in HCC *vs*. inflamed livers is shown in **Fig 1B**. The full list of enrichments using two different approaches is reported in **S3 Table** (David GO analysis) and **S4 Table** (Revigo clusters). The top-ranking categories of up-regulated genes in inflamed livers were related to cell adhesion, migration, organization of the extracellular matrix and actin cytoskeleton. Some notable genes related to cell adhesion and cytoskeleton organization included many collagen genes (*Col3a1* and *Col4a5* among many others), cadherin-1 (*Cdh1*) and matrix metalloproteinase-2 (*Mmp2*) (**Fig 1C** and **1D**). In addition, nearly all enzymes involved in glutathione metabolism were among the most up-regulated genes in inflamed livers, including glutamate-cysteine ligase (*Gclc*), the first and rate-limiting enzyme of glutathione synthesis, several glutathione S-transferases (*Gstt2* and *Gsta2* among the others), glutathione peroxidases (such as *Gpx4*) and the main transcriptional regulators of antioxidant genes, *Nfe2l2* (encoding for NRF2) and its dimerization partner *Mafk* (**Fig 1C** and **1D** and **S2 Table**). These observations are consistent with the notion that chronic portal inflammation in *Mdr2^-/-^* livers results in increased oxidative stress as well as connective tissue deposition, leading to progression to fibrosis [9,13]. More interestingly, while all genes involved in fibrosis were selectively down regulated in the transition from inflammation to cancer, the antioxidant response was further upregulated in liver cancers (**Fig 1B** and **1D**).

GO categories associated to genes down-regulated in inflamed livers were mainly related to oxidation-reduction processes (**Fig 1B**). Specifically, genes encoding hydroxylases involved in bile acid biosynthesis (such as the critical enzymes *Cyp7b1* and *Cyp8b1*) were downregulated as part of the negative feedback exerted by the excess of non-neutralized bile acids (**S2 Table**). Several Phase I enzymes belonging to the cytochrome p450 (CYP450) superfamily (*Cyp2a5*, *Cyp2c39*, and *Cyp4a32* among many others) and their main transcriptional regulator, the hepatocyte-specific nuclear receptor Constitutive Androstane Receptor (CAR, encoded by *Nr1i3*) [14,15] were also downregulated in inflamed livers (**Fig 1D** and **1C**). Phase I enzymes act by hydroxylating hydrophobic xeno- and endobiotics (including hydrophobic bile acids), thus increasing their solubility and reducing their toxicity. Interestingly, the same group of genes was strongly up-regulated in cancer nodules together with classical liver cancer markers (*Afp*, encoding alpha-fetoprotein among many others) (**Fig 1C** and **1D**) [16,17].

Overall, transcriptomic analyses indicate that the massive inflammatory and fibrotic response occurring in inflamed *Mdr2^-/-^* livers is reduced in tumor nodules, concurrently with the upregulation of hydroxylases of the CYP450 family.

### Overexpression of detoxification enzymes in *Mdr2^-/-^* cancers

**Fig 2:**
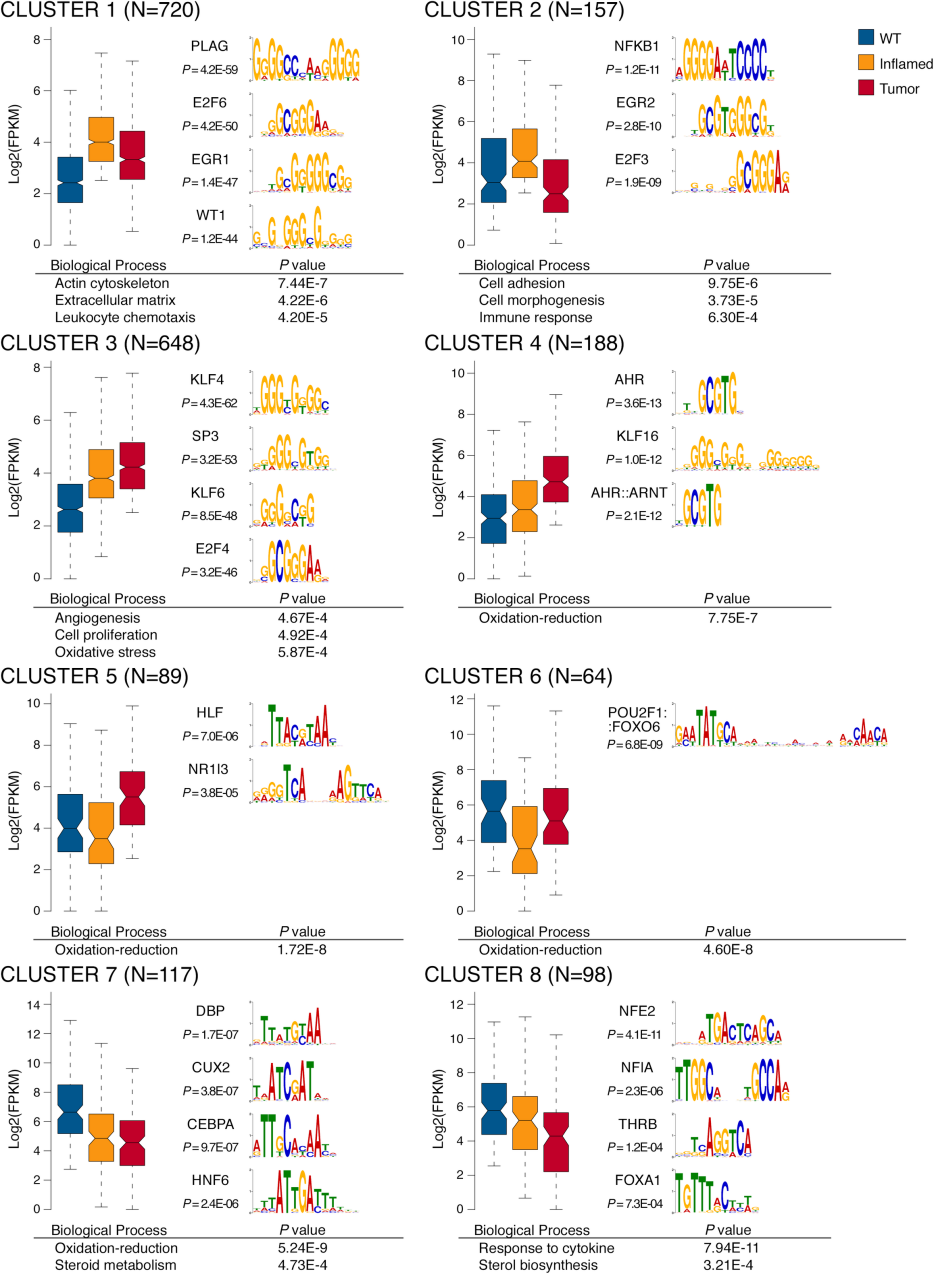
Stage-specific gene expression profile of *Mdr2^-/-^* livers. Clusters have been generated by considering differentially expressed genes with FC ≥2, q-value ≤0.05, FPKM ≥2. The number of genes per cluster is indicated in each plot. Bottom panels report selected GO terms enriched for each cluster. Right panels show PWMs overrepresented on the promoters (+/-1000 bp from TSS) of genes of each cluster, whose cognate TF expression trend is consistent with the cluster.

In order to better discriminate groups of genes with distinct behaviors during tumorigenesis, we divided differentially expressed genes into 8 clusters (**S5 Table**) based on their transcriptional profiles in the two disease stages considered (**Fig 2**, left panels). Next, to identify the transcription factors (TF) that selectively control gene expression in each cluster, we determined the TF consensus DNA binding sites (described by position weight matrixes, PWMs) that were statistically overrepresented in the promoters of the differentially expressed genes relative to a background including the promoters of all Ensembl-annotated genes (± 1000 bp relative to their transcription start sites) [18]. An initial list of candidate regulatory TFs was generated based on the statistical over-representation of the cognate DNA recognition motif and then filtered based on the expression of each TF (**Fig 2**, right panels). We also performed GO analysis of genes differentially expressed within these 8 clusters and identified the associated enriched GO terms (**Fig 2**, bottom panels). The full list of GO categories and PWMs associated to each cluster is available in **S6** and **S7 Tables**.

Overall, cluster 1, 2 and 3 included genes whose expression was increased in inflamed livers. While the expression of genes associated with fibrosis and inflammation (cluster 1 and 2) decreased in tumor nodules, genes involved in oxidative stress responses, angiogenesis and cell proliferation (cluster 3) were all further up-regulated in the transition from inflammation to cancer. To identify the TFs selectively involved in the transition between inflamed and tumor livers, we focused our attention on the genes that were selectively up-regulated at the tumor stage (cluster 4, 5 and 6). Interestingly, this subset of genes was homogeneously related to oxidation-reduction processes, notably those catalyzed by CYP450 family hydroxylases and involved in mono-oxygenation and detoxification of hydrophobic substances. Within the same clusters, the most over-represented DNA binding motifs included those for CAR (Constitutive Androstane Receptor, encoded by *Nr1i3*), AHR (Aryl Hydrocarbon Receptor) and its dimerization partner ARNT (AHR Nuclear Translocator), three TFs significantly overexpressed at the HCC stage (**S2 Table**).

Overall, these data indicate that the transition from inflammation to cancer is associated with the increased expression of genes involved in detoxification of xeno- and endobiotics such as bile acids [14].

**Fig 3:**
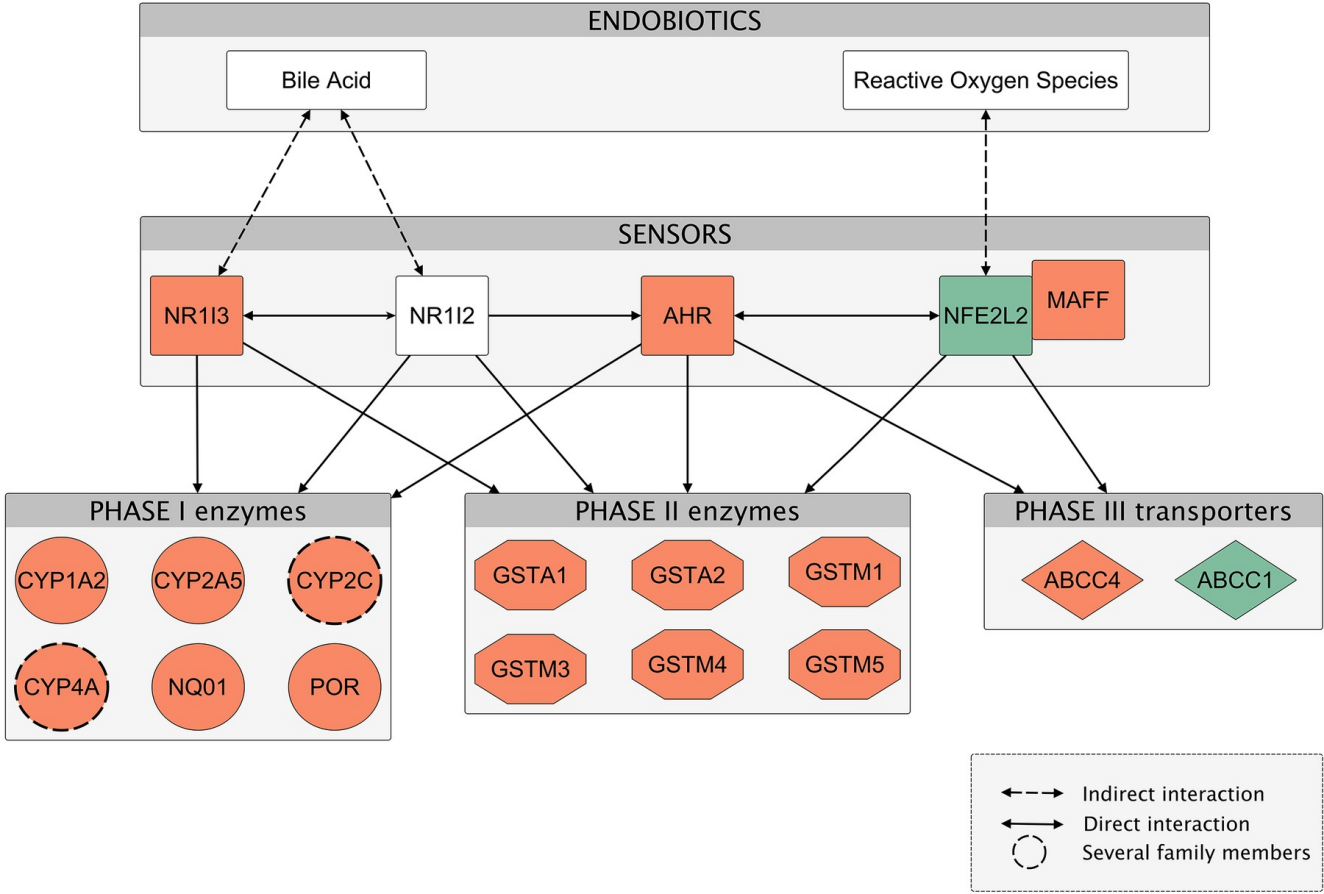
*Mdr2^-/-^* tumor development is characterized by global overexpression of endobiotics detoxification pathway members. Ingenuity pathway analysis was used to generate the network overlaid with relative gene expression levels of inflammation to tumor transition. Node colors indicate the upregulated (red) and downregulated (green) genes relative to the comparison of tumor samples over inflamed livers. Items depicted by a dashed circle represent several members of the *Cyp2c* (specifically, *Cyp2c29*, *Cyp2c37*, *Cyp2c38*, *Cyp2c39*, *Cyp2c40*, *Cyp2c44*, *Cyp2c50*, *Cyp2c55*, *Cyp2c67*, *Cyp2c68*, *Cyp2c69*, *Cyp2c70*) and *Cyp4a* (*Cyp4a10*, *Cyp4a12a*, *Cyp4a12b*, *Cyp4a14*, *Cyp4a32*) families. Network edges (the relationship between nodes represented by lines and arrows) represent direct (solid lines) and indirect (dashed lines) interactions between molecules as supported by information in the Ingenuity knowledge base. Each functional class of molecules is represented by a different node shape.

To gain insight into the functional consequences of transcriptional changes occurring in the transition from inflammation to tumors, we used Ingenuity Pathway Analysis (IPA). IPA was used to assemble a network based on genes differentially expressed between inflamed and tumor samples (**Fig 3**). This analysis revealed that tumor development was characterized by a global up regulation of members of the endobiotics detoxification pathways. Specifically, toxic bile in *Mdr2^-/-^* livers induced the expression of CAR *(Nr1i3)*, which regulates Phase I enzymes (*Cyp1a2*, *Cyp2a5*, *Cyp2c* and *Cyp4a* families, *POR*, *NQO1*), Phase II enzymes involved in conjugation of toxic agents (such as several glutathione S-transferases) and Phase III transporters (*Abcc4/Mrp4*, which mediates the cotransport of reduced glutathione with bile acids, thus enhancing their detoxification) [14,19]. It is important to note that also PXR *(Nr1i2)*, whose expression was increased in inflamed liver (cluster 3 in **Fig 2**), is activated by bile acids, in particular by the highly toxic lithocholic acid (LCA) [20] and that it also regulates Phase I and II enzymes and Phase III transporters. In the transition from inflammation to tumors NRF2 *(Nfe2l2)* was downregulated but the antioxidant pathway was maintained upregulated probably by the xenobiotic receptors CAR, PXR and AHR.

We next reanalyzed previous whole exome sequencing (WES) and whole genome sequencing (WGS) data we generated using *Mdr2^-/-^* cancers [11] in order to determine whether genes encoding enzymes involved in bile acid detoxification undergo amplification events. We found that a consistent number of genes (n = 44) involved in endobiotics detoxification pathway were amplified in 8 out of 10 sequenced samples (**S5 Fig**).

Overall, these data suggest the existence of a selective pressure favoring the overexpression of genes encoding enzymes that control detoxification of bile acids and that in some cases this can be achieved by gene amplification.

### Differential usage of genomic regulatory information in inflamed and neoplastic livers

**Fig 4:**
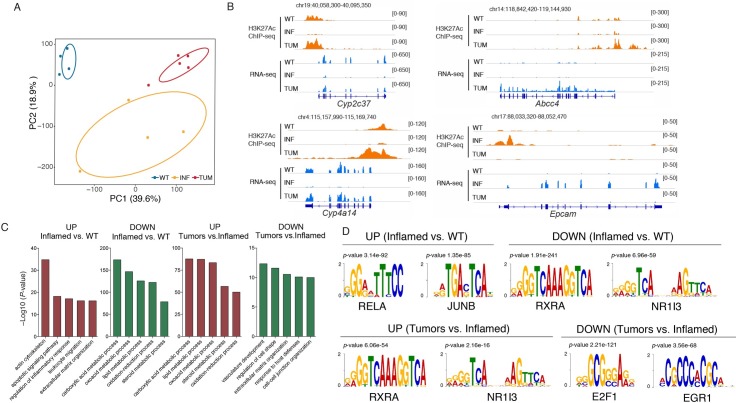
H3K27Ac profiling of *Mdr2^-/-^* samples. H3K27Ac ChIP-seq were performed on samples from normal (WT), inflamed (INF) and HCC (nodules) livers (TUM) from mice treated with clodronate liposomes. **(A)** PCA analysis. 2 out of 15 samples were removed as outliers. **(B)** GO categories associated with differentially acetylated genomic regions of WT, inflamed and liver samples, as inferred from a GREAT analysis. **(C)** Snapshots of representative WT, inflamed and tumor samples showing H3K27Ac and RNA-seq data of differentially expressed genes. **(D)** Motif finding analysis of the genomic regions analyzed in (B). The PWMs indicated have been filtered based on the expression of TFs on each group.

To obtain a more detailed view of the gene regulatory networks underlying adaptive changes in gene expression during tumorigenesis in the *Mdr2^-/-^* livers, we performed H3K27Ac ChIP-seq on the same fifteen samples (five per experimental group) used for RNA-seq profiling. Histone H3 Lysine 27 (H3K27) acetylation is a histone modification deposited at gene promoters and at enhancers when they are bound by activating TFs, and is thus informative of the activity state of these *cis*-regulatory elements [21–23]. Based on the H3K27Ac profiles, WT liver, inflamed *Mdr2^-/-^* livers and cancers clustered separately (**Fig 4A**), indicating a differential usage of the genomic *cis*-regulatory information.

To gain insight into the functional responses controlled by *cis*-regulatory regions selectively acetylated in inflamed livers and then in cancers, we used the GREAT tool [24]. GREAT links sets of genomic regions to putative biological functions based on the functional annotations of the nearby genes, with a score that takes into account the distance between regions and genes and therefore the likelihood of correct assignment. Genomic regions specifically activated in inflammation were enriched in functional terms related to extracellular matrix organization, as well as with leukocyte migration, a result consistent with the inflammatory and fibrotic response identified by gene expression profiling at the same stage (**S8 Table** and **Fig 4B**). Coherently with transcriptomic data, the genomic regions associated to these fibrotic functional terms were deacetylated in the transition from chronic inflammation to cancer, together with regions associated to developmental functions. The transition to inflammation was also characterized by a repression of acetylated regions associated with liver metabolic function (oxidation-reduction process). Interestingly, the oxidation-reduction processes terms were retrieved on regulatory regions upregulated in the comparison between nodules and inflammation (**Fig 4B**). Snapshots of three representative samples (**Fig 4C**) show tumor-specific increased acetylation and RNA expression of *Cyp2c37* and *Cyp4a14*, encoding Phase I detoxification enzymes, and *Abcc4*, encoding a bile acid transporter. Conversely, the H3K27Ac signal on the promoter of the *Epcam* gene, encoding the cell adhesion molecule, was induced in inflamed livers and subsequently repressed in tumors.

Next, we determined the TF consensus DNA binding sites that were statistically overrepresented in the differentially acetylated *cis*-regulatory regions [18]. In line with the GREAT analysis, motifs for TFs involved in the inflammatory and fibrotic response (including NF-kB and AP-1) were enriched in regions that gained acetylation in inflamed livers relative to normal controls, (**Fig 4D** and **S9 Table**). More importantly, when analyzing motifs over-represented in cancers relative to inflamed livers, we identified the DNA binding site for CAR (*Nr1i3*, belonging to the NR1 family of Thyroid hormone receptor-related factors), which is also overexpressed in *Mdr2^-/-^* liver cancers.

Overall, these results are in line with the transcriptional profiling datasets discussed above and suggest that the increased activity of a CAR-activated detoxification pathway occurs (and might be functionally involved) in the transition from inflammation to cancer.

### Concentration of toxic bile acids is reduced to normal levels in HCC

**Fig 5:**
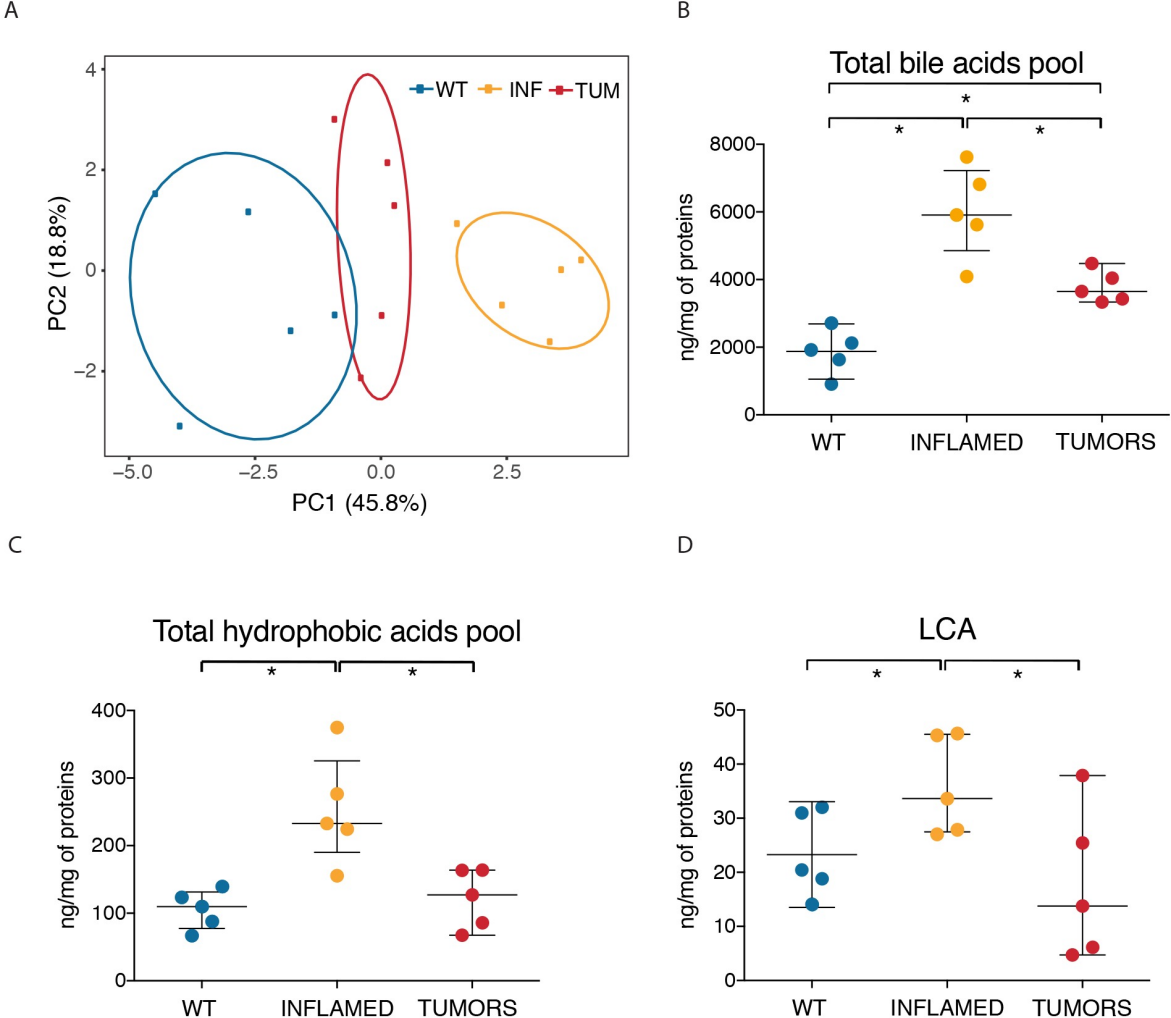
Toxic bile acids are reduced to normal levels in HCC stage. Bile acid quantification of *Mdr2^-/-^* livers by LC-MS/MS analysis. Bile acids were extracted from macrophage-depleted livers from 15/17-month-old *Mdr2^-/-^* mice (HCC stage), 8-month-old *Mdr2^-/-^* mice (inflamed stage) and age matched FVB/NJ mice (WT control) and quantified by MS. Each dot of the beeswarm plots (panel B, C and D) represents the sum of all bile acids quantified in one liver sample (5 animals per groups). Values of each quantified bile acid are listed in Table 1. The central black bars indicate the median with the 1st and 3rd quartile. In all analyses, differences were assessed using Welch Two Sample t-test (p < 0.05). Data are shown as nanogram of bile acids on mg of proteins extracted. **(A)** PCA of total bile acids plotted in B **(B)** Sum of total bile acids concentration, **(C)** Sum of hydrophobic bile acids, **(D)** LCA.

The upregulation of CAR expression in HCC as well as the over-representation of CAR motifs in the *cis*-regulatory elements that were selectively hyper-acetylated in cancers as compared to inflamed livers, suggest the hypothesis that transformed hepatocytes have acquired the ability to efficiently detoxify bile acids, which in turn would explain their ability to survive and proliferate in the context milieu of *Mdr2^-/-^* livers. To directly address this possibility, we used HPLC separation coupled to tandem mass spectrometry (HPLC-MS/MS) to quantify the concentrations of free bile acids and their taurine and glycine conjugates in the same liver samples used for expression and epigenetic analyses. 15 different species of bile acids were unambiguously identified and quantified (**Table 1**). PCA analysis showed that wild type, inflamed and tumor samples could be clearly separated from each other and that tumor samples were more similar to wild type controls than to inflamed livers (**Fig 5A**). Total bile acid levels were significantly increased in inflamed livers compared to age-matched controls (**Fig 5B**). More interestingly, tumor samples were characterized by a significant decrease (*p* ≤ 0.05) of total bile acids levels, which however did not return to the same values as those measured in the normal tissue. The observed decrease is not due to an impaired bile acids synthesis by tumor cells, as the expression of the rate-limiting enzymes *Cyp7a1* and *Cyp27a1* was not affected in the transition from inflammation to tumors. When only hydrophobic bile acids (namely, those with the highest cytotoxic potential) were considered, tumor livers showed a robust decrease in their levels, that returned to those measured in the matched wild type controls (**Fig 5C** and **Table 1**). In particular, the most hydrophobic and toxic bile acid, lithocolic acid (LCA) decreased to median levels that were even lower than those measured in normal livers (**Fig 5D**).

**Table 1:** Bile acids content in *Mdr2^-/-^* livers.

			average ng/mg of protein lysate		
-OH groups			Normal (n = 5)	Inflamed (n = 5)	HCC (n = 5)
3	**TMCA(α+β)**	tauromuricholic acid	985.04 ± 371.07	3716.44 ± 858.25	2380.62 ± 414.59
3	**MCAω**	muricholic acid omega	1.22 ± 1.42	17.85 ± 9.29	4.39 ± 4.32
3	**MCAβ**	muricholic acid beta	12.05 ± 9.9	150.78 ± 68.91	73.66 ± 33.75
3	**MCAα**	muricholic acid alpha	1.05 ± 0.99	6.12 ± 2.11	2.61 ± 1.64
3	**GCA**	glycocholic acid	2.14 ± 1.05	3.79 ± 0.88	1.68 ± 0.44
3	**TCA**	taurocholic acid	730.99 ± 265.15	1831.86 ± 380.33	1168.44 ± 122.87
3	**CA**	cholic acid	36.44 ± 17.69	32.93 ± 17.93	34.56 ± 25.39
2	**TUDCA**	tauroursodeoxycholic acid	33.64 ± 11.97	93.52 ± 54.12	25.82 ± 15.57
2	**UDCA**	ursodeoxycholic acid	5.37 ± 1.3	5.36 ± 0.9	6.54 ± 4.07
2	**TCDCA**	taurochenodeoxycholic acid	24.56 ± 10.69	110.93 ± 37.69	60.84 ± 23.92
2	**HDCA**	hyodeoxycholic acid	1.39 ± 0.78	1.32 ± 0.31	1.32 ± 0.62
2	**CDCA**	chenodeoxycholic acid	7.25 ± 6.04	0.63 ± 0.44	1.85 ± 0.59
2	**DCA**	deoxycholic acid	3.54 ± 1.51	1.24 ± 0.53	3.2 ± 1.62
1	**TLCA**	taurolithocholic acid	6.44 ± 3.27	3.95 ± 0.92	4.41 ± 0.72
1	**LCA**	lithocholic acid	23.28 ± 7.87	35.93 ± 9.12	17.6 ± 14.01

Overall, these data demonstrate that the total pool of bile acids, and even more so the hydrophobic and toxic ones, were substantially reduced in the transformed hepatocytes of *Mdr2^-/-^* tumors, indicating that the transition between the inflammatory stage and tumors involves an increased ability to detoxify and dispose of these endobiotics.

### Impact of CAR inhibition on *Mdr2^-/-^* tumors

**Fig 6:**
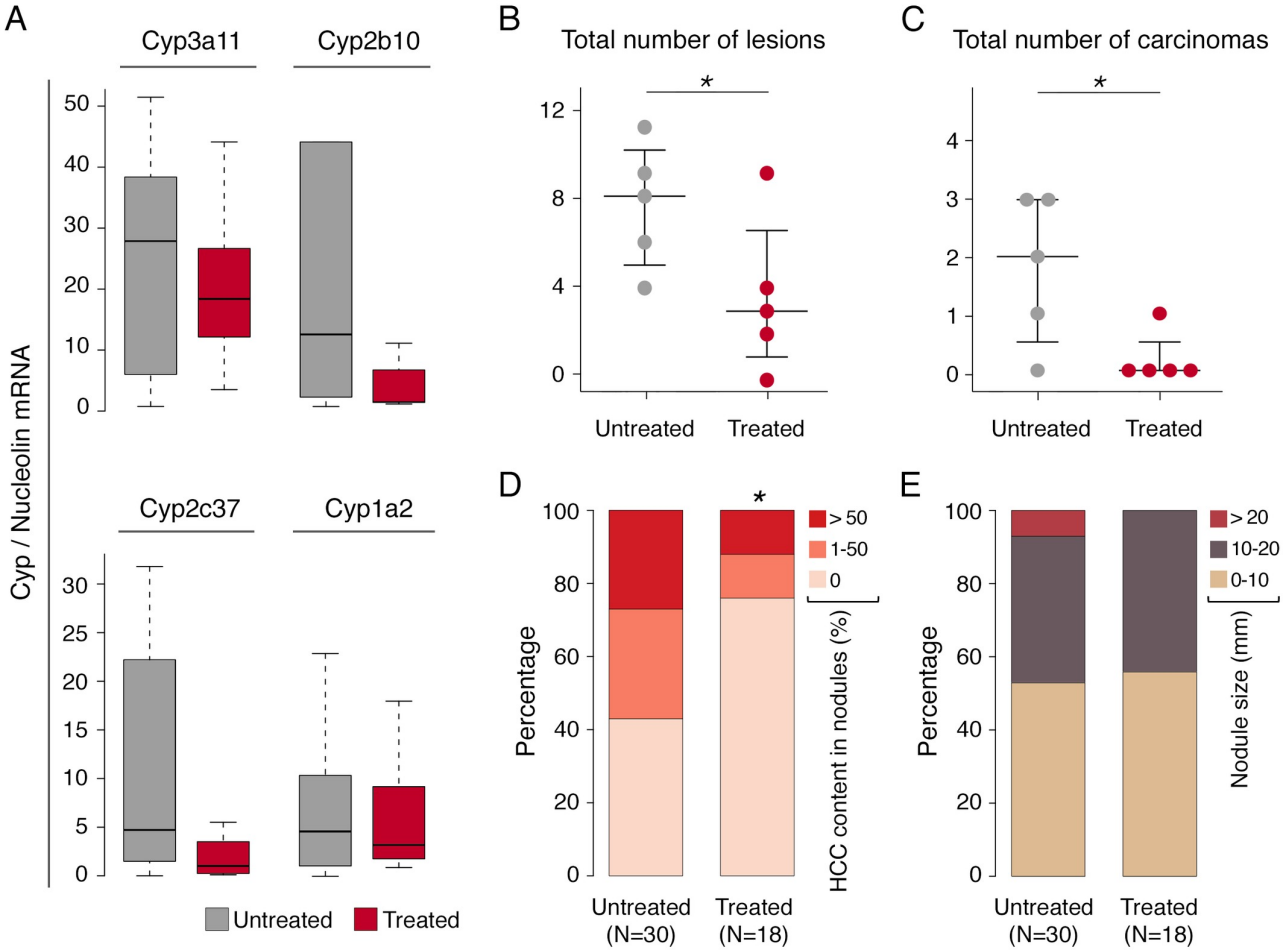
CAR inhibition blocks cancer progression in *Mdr2*-KO livers. Nodules from HCC livers of *Mdr2^-/-^* mice vehicle (UT) or CAR inhibitor treated (T) (50 mg/kg) were measured by caliper, collected for histological analysis and for RT-QPCR expression (see also S10 Table). **(A)** Q-PCR analysis of representative CAR target genes (UT, nodule number = 22; T, nodule number = 12). **(B)** Total number of lesions per mouse. **(C)** Total number of carcinomas per mouse. The central black bars indicate the median. **(D)** Tumor content measured as a percentage of HCC in each nodule. The number of nodules in the two groups are reported in parentheses. **(E)** Size differences in nodules from treated and untreated *Mdr2^-/-^* mouse groups. In all analyses, differences were assessed using one-sided Mann-Whitney test (p < 0.05).

Finally, we set out to investigate whether CAR inhibition might impact viability of tumors in *Mdr2^-/-^* mice. To this aim, we randomized 15 months-old *Mdr2^-/-^* mice (5 animals per group) to receive either 5α-androstan-3α-ol, a selective CAR inhibitor [25] or vehicle only via intraperitoneal injection. After 2 weeks of CAR inhibitor treatment and 48 hours of clodronate treatment to deplete macrophages, mice were sacrificed and tumors from the two cohorts were compared in terms of nodule number, size, histology and tumor content (**S10 Table**). Alanine aminotransferase (ALT) and aspartate aminotransferase (AST) levels were determined in plasma samples collected from vehicle and CAR inhibitor-treated mice to determine the possible occurrence of general hepatotoxicity. ALT and AST plasma levels were constitutively elevated in *Mdr2^-/-^* mice but not further increased following treatment (**S6 Fig**). Expression of representative CAR targets measured by quantitative RT-PCR on nodules from treated and untreated mice (such as *Cyp3a11*, *Cyp2b10*, *Cyp2c37 and Cyp1a2*) [15], was repressed by the CAR inhibitor, thus indicating efficient CAR inhibition in treated mice (**Fig 6A**).

Treated animals showed a significantly lower number of neoplastic lesions (adenomas and carcinomas) per mouse when compared with the untreated cohort (**Fig 6B** and **S7 Fig**). When only carcinomas were considered, treated mice showed a significant depletion in HCC nodules (**Fig 6C**), while adenomatous nodules (containing no HCC foci) were over-represented (**Fig 6D**). In addition, no mouse treated with CAR inhibitor had nodules bigger than 20 mm (**Fig 6E**).

Altogether, inhibition of CAR activity had an overall negative impact on tumor progression and on existing cancer nodules.

## Discussion

A large body of evidence indicates that chronic inflammation is associated with many cancer types but it is still unclear how cancer cells become able to survive within the cytotoxic microenvironment associated with high local concentrations of some inflammatory agents. In this study, we set out to dissect the epigenetic and transcriptional changes occurring in the transition from chronic inflammation to tumors in the specific context of the liver carcinogenesis in the *Mdr2^-/-^* mouse. Although this mouse is traditionally considered a paradigmatic model of inflammation-induced cancer, it is genetically and etiologically different from common types of human HCC and it is more appropriate to deem it representative of those tumors that develop in the context of a highly toxic microenvironment that determines a vicious circle of cell death and regeneration. The main finding of our study is that for tumors to develop in such a context, it is necessary that transcriptional and genetic changes occur that equip parenchymal cells with the ability to resist toxicity exerted by micro-environmental components.

By gene expression and epigenomic profiling, we found that HCC livers are characterized by CAR-mediated activation of Phase I and II detoxification pathways and transporters, which promote the detoxification and excretion of toxic bile acids that accumulate in livers of *Mdr2^-/-^* mice and cause the extensive inflammation typical of the precancerous stage.

Elimination of bile acids includes Phase I reactions (mainly consisting in the hydroxylation of bile acids and in a consequent reduction of their hydrophobicity) and Phase II reactions consisting in the conjugation of bile acids with molecules that further increase their hydrophilicity, thus reducing toxicity and enabling urinary excretion [26]. In cholestatic diseases, Pregnane X Receptor (PXR, *Nr1i2*) and the Constitutive Androstane Receptor (CAR, *Nr1i3*) represent the two key nuclear receptors controlling the expression of enzymes and transporters involved in bile detoxification and excretion in hepatocytes [27]. We found that PXR expression is induced already in inflamed livers and remains high in HCC. This effect might be the result of PXR activation by LCA, a well know direct ligand of PXR [20], which we found increased in inflamed livers. Interestingly, the appearance of cancer nodules in the inflamed livers is characterized by a transcriptional upregulation of the two other main regulators of xenobiotic metabolism, namely AHR and CAR. As a result, the majority of genes involved in Phase I and Phase II detoxification pathways are strongly upregulated in cancers. Consistently, the increased expression of xenobiotic receptors is paralleled by the upregulation of genes with overrepresented xenobiotic receptors (XR) motifs in their promoters. It is also interesting to note that tumors developing in *Mdr2^-/-^* mice show a very low burden of DNA mutations but massive gene amplification and rearrangements at late cancer stages [8,11]. Genes encoding components of the xenobiotic detoxification pathways are indeed amplified themselves, which is consistent with a selective advantage provided by their increased expression.

Overall, these results indicate that the accumulation of toxic hydrophobic bile acids, and in particular LCA, during chronic inflammation activates a detoxification program that is initially insufficient to prevent cellular damage and the vicious cycle of death and regeneration that characterizes the long pre-tumoral stage of liver disease. It is only a further increase in the activity of the detoxification program controlled by xenobiotic receptors that enables hepatocytes to undergo uncontrolled proliferation, likely stimulated by the chronic inflammatory environment. In this regard, CAR has been shown to be activated by LCA *in vivo* and other bile acids were found to activate the ligand binding domain of CAR *in vitro* [15,28]. The secondary bile acid LCA is the most potent cholestatic agent and causes liver damage unless it is efficiently eliminated [29]. Notably, CAR has been shown to have a fundamental protective role in the response to LCA *in vivo* since CAR-KO mice have more severe defects in LCA detoxification compared to PXR-KO mice [15] and CAR activation in transgenic mice confers resistance to the hepatotoxicity of LCA [30].

Importantly, our results indicate that CAR inhibition arrests tumor progression in that it reduces the number of bigger lesions with high HCC content. Although it was not feasible to test CAR inhibitor during the transition from inflammation stage to early adenoma (a six months-long process), it is tempting to speculate that pharmacological inhibition of CAR may be useful also to block HCC onset. Consistently with our findings, chronic CAR activation has been shown to result in liver carcinogenesis, as CAR-KO mice are completely resistant to tumorigenic effects of chronic xenobiotic stress [31] and long-term activation of CAR and β-catenin induces liver tumorigenesis [32].

Overall, our study points to a general framework for tumorigenesis occurring in the context of toxic micro-environments that may extend to other cases such as tumors associated with chronic exposure to noxious chemicals. Specifically, conditions that induce a stress response program also increase the selective pressure on pre-neoplastic cells to develop powerful mechanisms to cope with the same stress, as also indicated by the frequent amplification of genes encoding components of the endo/xenobiotic detoxification pathways in *Mdr2^-/-^* HCC. These data also suggest the possibility to use prophylactic or therapeutic approaches targeting xenobiotic receptors in such contexts.

## Materials and methods

### Ethics statement

Experiments involving mice have been carried out in accordance with the Italian Laws (D.L.vo 116/92) which enforces the EU 86/609 directive. The Ministry of Health was notified of this project in March 2014 (Project number: 02/2014).

### Murine liver tissues preparation and histology

Founders of the FVB.129P2-Abcb4^tm1Bor^ (*Mdr2^-/-^*) and FVB/NJ (*Mdr2*-WT) mice were purchased from The Jackson Laboratory. Colonies of both strains were maintained under specific pathogen-free conditions. Mice (both males and females) were treated with liposomes loaded with 5 μg clodronate or with PBS (www.clodronateliposomes.com) via tail vein injection, 48h prior to sacrifice. Each nodule or liver tissue sample was partly being snap frozen for DNA/RNA/protein extraction. Furthermore, a portion of each specimen was histologically assessed after overnight fixation in 4% formaldehyde and paraffin inclusion.

### Immunohistochemistry and histopathological examination

Anti-IBA1 immuno-stains were performed on 4 μm sections. After de-waxing and re-hydration in ethanol, antigen de-masking was done in sodium citrate buffer in a water bath at 95°C for 45 minutes. Endogenous peroxidases were quenched with a 5 min treatment in 3% H_2_O_2_. Slides were incubated with rabbit IBA1 antibody (Wako, 019–19741) diluted 1:500, and developed with HRP polymer (DAKO). Slides were finally counterstained with hematoxylin and mounted with Eukitt. The histological classification of hepatocellular proliferative lesions was performed according to Thoolen et al. [33]. For each mouse, either the composition of the tumor (in terms of percentage of adenoma and/or carcinoma) or the number of hepatocellular adenomas, early carcinomas (defined as adenomas containing focus of arising carcinoma), carcinomas, and the total number of neoplastic lesions were evaluated by a mouse pathologist. Samples were coded without reference to experimental group and examined blindly.

### ALT and AST assay

Blood samples were incubated on ice for 30 min to coagulate and were centrifuged for 10 min at 5000 rpm to separate the serum. Colorimetric determination of ALT levels was performed using TECO Diagnostics assay kits (Teco Diagnostics, Anaheim, CA). Procedures were performed as described by the manufacturer, except for a proportional decrease in volume to minimize the use of serum per assay. Colorimetric determination of AST levels was performed in the diagnostic laboratory of Humanitas Clinical and Research Center.

### RNA sequencing

RNA-seq was carried out using previously described protocols [34] on an Illumina HiSeq2000 platform. Frozen tissue samples were homogenized with a dounce homogenizer or with gentleMACS Dissociator (Miltenyi Biotec), depending on the tissue volume. Total RNA was extracted using Maxwell 16 LEV SimplyRNA cells kit (Promega) and run on Agilent Bioanalyzer 2100 to assess sample integrity. mRNA-seq library preparation from 4 μg of total RNA was performed with TruSeq RNA Sample Prep Kit V2 (Illumina) according to the manufacturer’s instructions.

### Chromatin immunoprecipitation (ChIP)

ChIP was carried out as previously described [34]. Briefly, 350 mg of liver/tumoral fixed tissue have been used for each ChIP. Chopped tissue samples were further homogenized with gentleMACS Dissociator (Miltenyi Biotec) prior to lysis. Homogenized tissues were processed with a two-step lysis protocol for cellular and nuclear membranes disruption, followed by chromatin shearing by sonication. Each lysate was then immunoprecipitated overnight with 5 μg of anti H3K27Ac antibody (Abcam, ab4729, [23]) prebound to 100 μl of G protein-coupled paramagnetic beads (Dynabeads). After beads washing, DNA was eluted and crosslink was reversed by overnight incubation at 65°C. DNA was then purified by Qiaquick columns (Qiagen) and quantified with PicoGreen (Invitrogen). ChIP validation by Q-PCR has been done on an Applied Biosystems 7500 Fast Real-time PCR system (SYBR Green, Applied Biosystems). ChIP DNA libraries were prepared as previously described [34], and sequenced on an HiSeq2000 with a 36bp single end setting (Supplementary materials).

### Inhibition of CAR

15 months-old *Mdr2^-/-^* mice were treated with 5α-androstan-3α-ol (Steraloids, Newport), a selective CAR inhibitor, as previously described [25]. The inhibitor was dissolved in a DMSO/corn oil solution and administered at 50 mg/kg by intra-peritoneal injection. Each mouse was treated every 48 h, and received a total of 6 treatments. Animals were finally sacrificed 48 h after the last inhibitor administration and after macrophage ablation by clodronate liposomes, and all detectable nodules were collected for histological analysis. Grossly detectable hepatic nodules were counted and measured with a caliper.

### Liver bile acid content

Bile acid content was evaluated from normal, inflamed and HCC livers. Liver bile acids were extracted by Folch method in presence of 5-alpha-cholestane as internal standard and subjected to HPLC-MS/MS analysis. The analyses were performed on an API-4000 triple quadrupole mass spectrometer (AB Sciex) coupled with a HPLC system (Agilent) and CTC PAL HTS autosampler (PAL System). A detailed description of the sample preparation and the subsequent MS analysis is provided in the Supplemental materials file.

### Computational methods

Short reads obtained from Illumina HiSeq2000 runs were analyzed as described [34]. Detailed computational methods are described in the Supplemental materials file.

Accession numbers. Raw datasets are available in the Gene Expression Omnibus (GEO) database (http://www.ncbi.nlm.nih.gov/geo) under the accession GSE80777, which comprises ChIP-seq data (GSE80775) and expression data (GSE80776).

## Supporting information

S1 Fig Analysis of the effects of macrophage depletion in wild type and *Mdr2^-/-^* inflamed livers and cancers. **A)** Representative sections of *Mdr2*-WT and inflamed and HCC *Mdr2^-/-^* livers, treated with clodronate or control PBS liposomes, and stained with IBA1 antibody to evaluate the presence of macrophages.
**B)** Histogram reporting the mean percentage of IBA1 positive area per field at 400x. Averages from 4 representative fields per specimen, taken from 5 mice per group.
**C)** Gene ontology analysis on the set of differentially expressed genes identified in WT livers treated with clodronate with respect to control WT livers.
(TIF)

S2 Fig Effects of clodronate treatment on liver inflammatory cells. **A)** Expression of key markers of macrophages (CD11b and F4/80), T cells (CD3), B cells (B220), neutrophils (Myeloperoxidase, MPO), dendritic cells (CD11c) and NK cells (CD335) was evaluated in clodronate and PBS treated livers. **B)** Representative sections of *Mdr2*-WT, inflamed and HCC *Mdr2^-/-^* livers, treated with clodronate or control PBS liposomes, and stained with CD3 antibody to evaluate the presence of T lymphocytes. **C)** Histogram reporting the mean number of CD3 positive cells per field at 400x. Averages from 4 representative fields per specimen, differences were assessed using t-test, (p < 0.005).
(TIF)

S3 Fig Dendrogram of expressed genes in wild type livers and *Mdr2^-/-^* inflamed livers and cancers as measured by RNA-seq from clodronate treated mice. (TIF)

S4 Fig Q-PCR validation of a set of representative genes belonging to different clusters of Fig 2. (TIF)

S5 Fig Genes involved in detoxification of xenobiotics are frequently amplified in *Mdr2^-/-^* liver tumors. Log2 ratios between normalized gene coverage in tumoral and reference DNA in nodule samples that underwent whole exome sequencing (WES) or whole genome sequencing (WGS) are reported. Amplified or deleted regions are highlighted in yellow. Data are from Iannelli et al., 2014.
(TIF)

S6 Fig Alanine aminotransferase (ALT) and aspartate aminotransferase (AST) levels in plasma of mice treated with placebo or CAR inhibitor. Note that one sample of treated mouse was excluded from the assay because of erythrocyte hemolysis.
(TIF)

S7 Fig **(A)** Representative images of livers from an untreated (UT) and a treated (T) *Mdr2^-/-^* mouse. Scale bar = 1cm. **(B)** Representative hematoxylin/eosin histologic sections of HCC and adenoma from untreated and treated livers. Scale bar = 100 um.
(TIF)

S1 Table Gene Ontology terms identified by DAVID and associated with differentially expressed genes by comparing clodronate-treated and untreated livers. The list of GO terms refers to data shown in S1C Fig.
(XLSX)

S2 Table Differentially expressed genes identified separately by comparing wild type livers with *Mdr2^-/-^* inflamed livers and inflamed livers with cancer nodules. (XLSX)

S3 Table GO terms identified by DAVID and associated with differentially expressed genes from the lists in S2 Table. (XLSX)

S4 Table GO terms identified by Revigo and associated with differentially expressed genes from the lists in S2 Table. (XLSX)

S5 Table Clusters of differentially expressed genes in normal (WT), *Mdr2^-/-^* inflamed and HCC livers. (XLSX)

S6 Table GO terms associated with clusters of differentially expressed genes reported in S5 Table. (XLSX)

S7 Table Over-represented transcription factor motifs in the promoters of differentially expressed genes reported in S5 Table. (XLSX)

S8 Table GREAT analysis of enriched GO categories performed on the H3K27Ac ChIP-seq data sets. (XLSX)

S9 Table Over-represented transcription factor motifs at differentially acetylated regions. (XLSX)

S10 Table Histopathological evaluation of livers from *Mdr2^-/-^* mice, untreated and treated with CAR inhibitor. (XLSX)
