# Supplementary figures and images for: Sustained activation of detoxification pathways promotes liver carcinogenesis in response to chronic bile acid-mediated damage
Source: PLoS Genet. 2018 May 7;14(5):e1007380. doi: 10.1371/journal.pgen.1007380 (PMC5957449; doi:10.1371/journal.pgen.1007380)

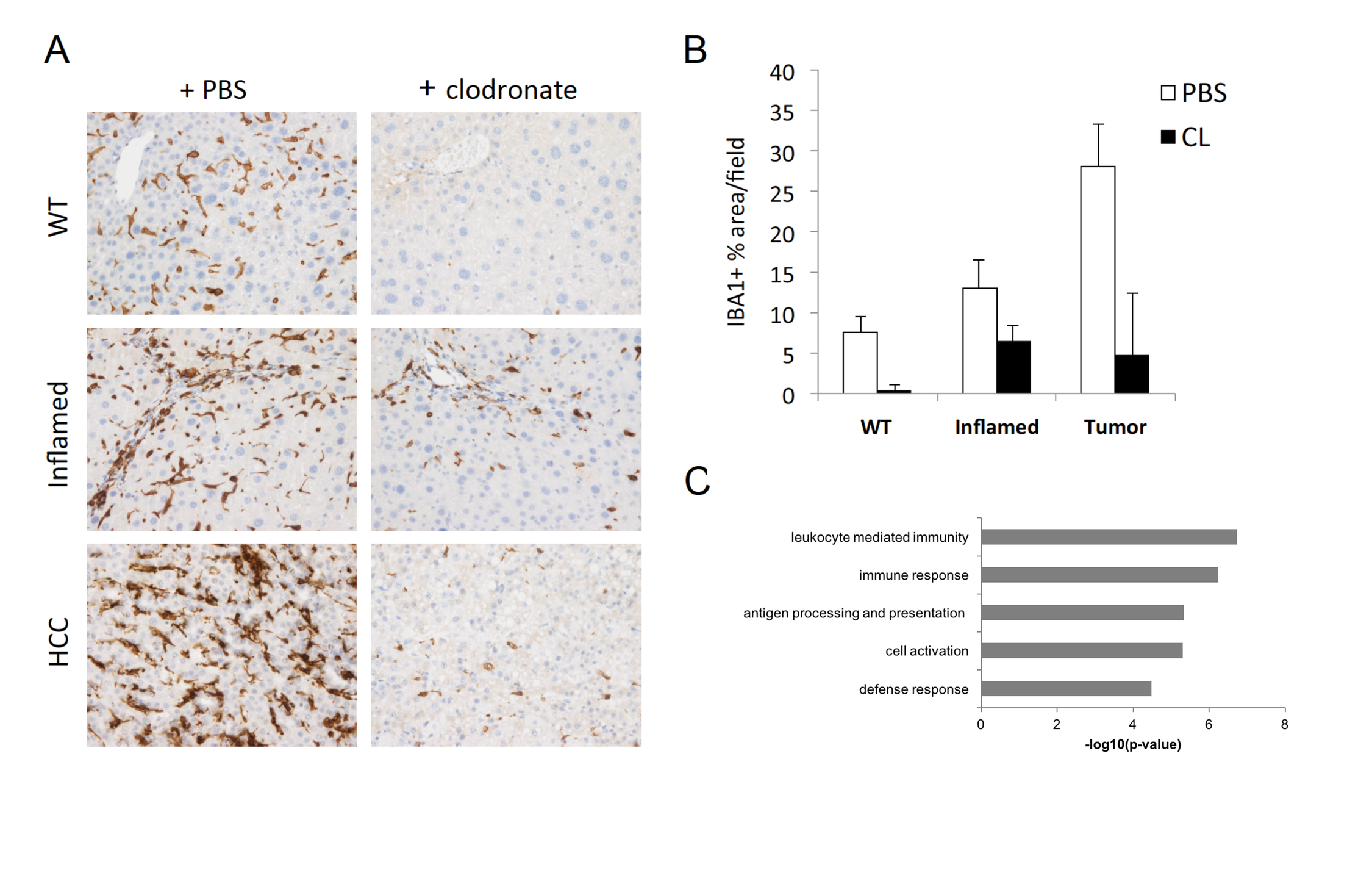

Supplement: S1 Fig — A) Representative sections of Mdr2-WT and inflamed and HCC Mdr2-/- livers, treated with clodronate or control PBS liposomes, and stained with IBA1 antibody to evaluate the presence of macrophages. B) Histogram reporting the mean percentage of IBA1 positive area per field at 400x. Averages from 4 representative fields per specimen, taken from 5 mice per group. C) Gene ontology analysis on the set of differentially expressed genes identified in WT livers treated with clodronate with respect to control WT livers. (TIF) [file pgen.1007380.s001.tif]

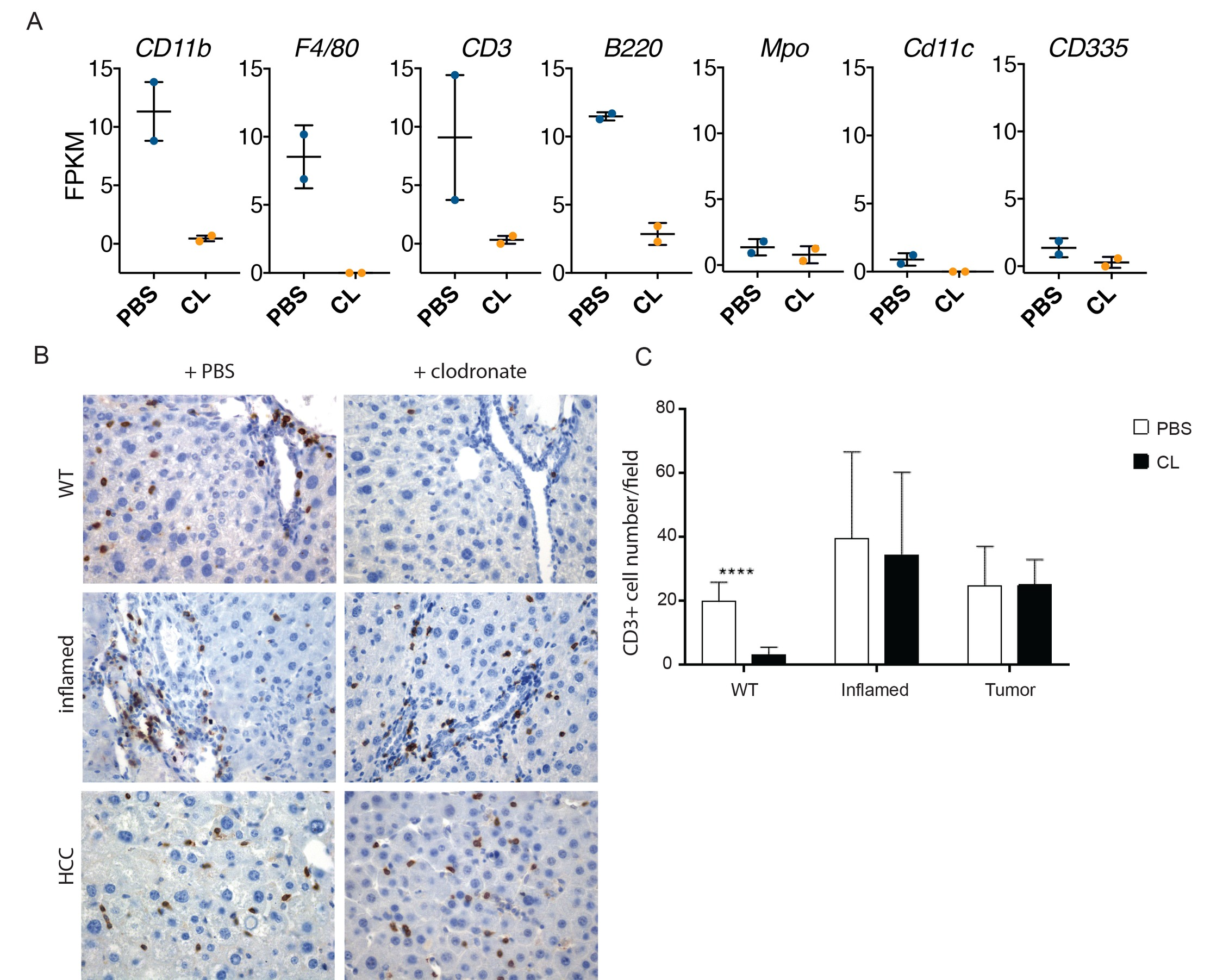

Supplement: S2 Fig — A) Expression of key markers of macrophages (CD11b and F4/80), T cells (CD3), B cells (B220), neutrophils (Myeloperoxidase, MPO), dendritic cells (CD11c) and NK cells (CD335) was evaluated in clodronate and PBS treated livers. B) Representative sections of Mdr2-WT, inflamed and HCC Mdr2-/- livers, treated with clodronate or control PBS liposomes, and stained with CD3 antibody to evaluate the presence of T lymphocytes. C) Histogram reporting the mean number of CD3 positive cells per field at 400x. Averages from 4 representative fields per specimen, differences were assessed using t-test, (p < 0.005). (TIF) [file pgen.1007380.s002.tif]

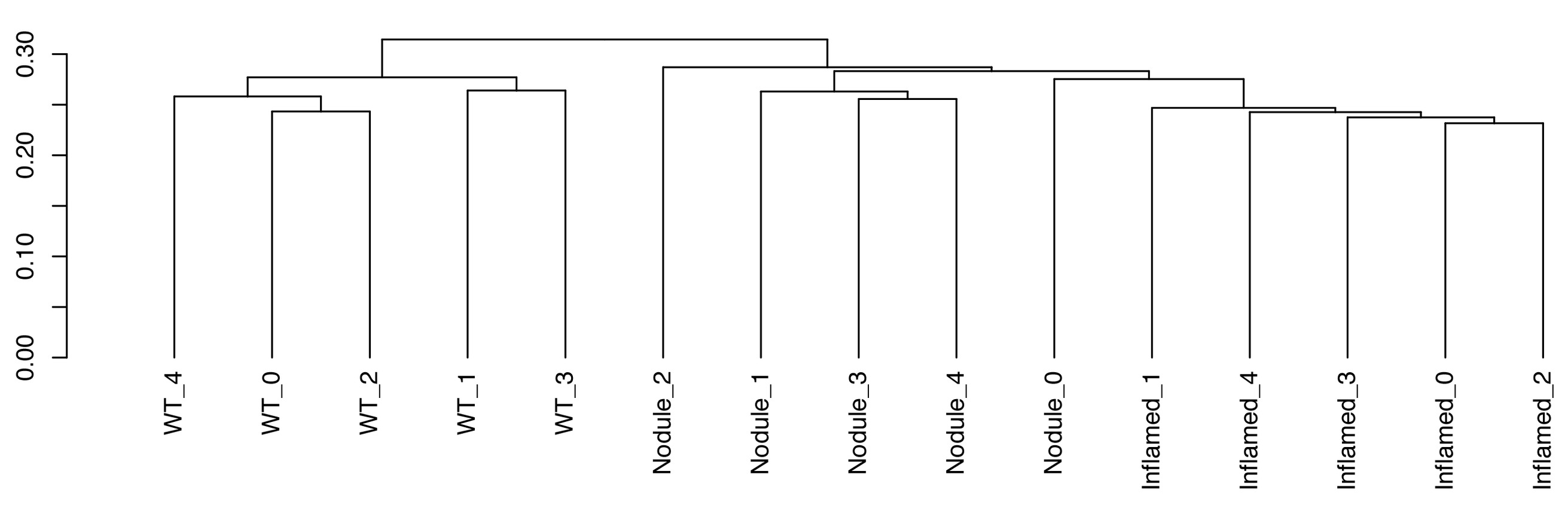

Supplement: S3 Fig — (TIF) [file pgen.1007380.s003.tif]

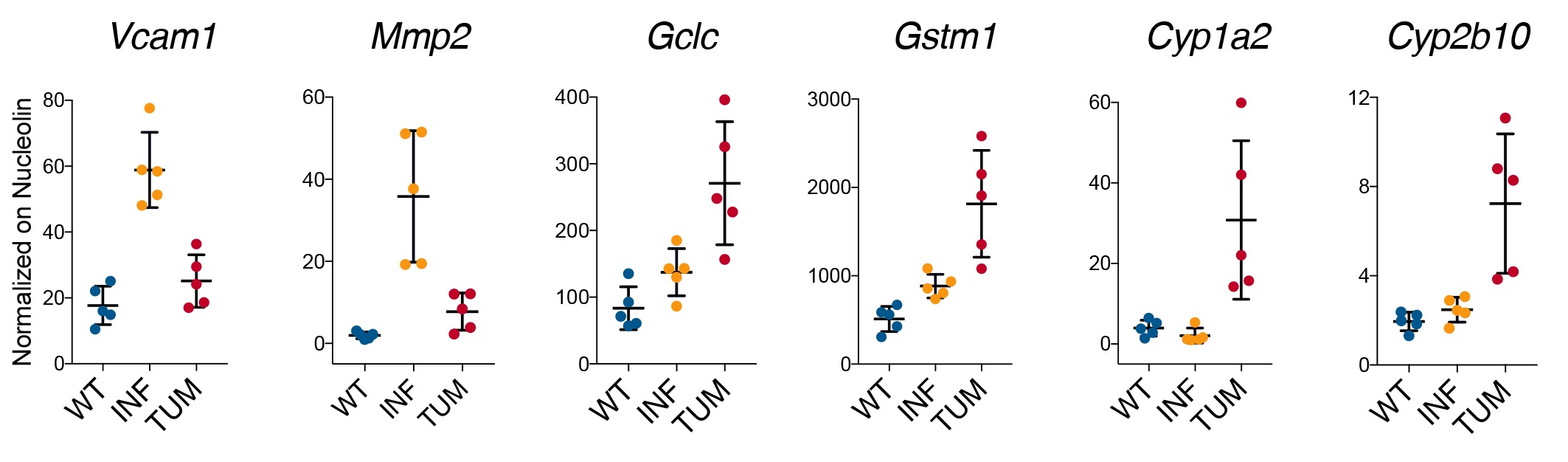

Supplement: S4 Fig — (TIF) [file pgen.1007380.s004.tif]

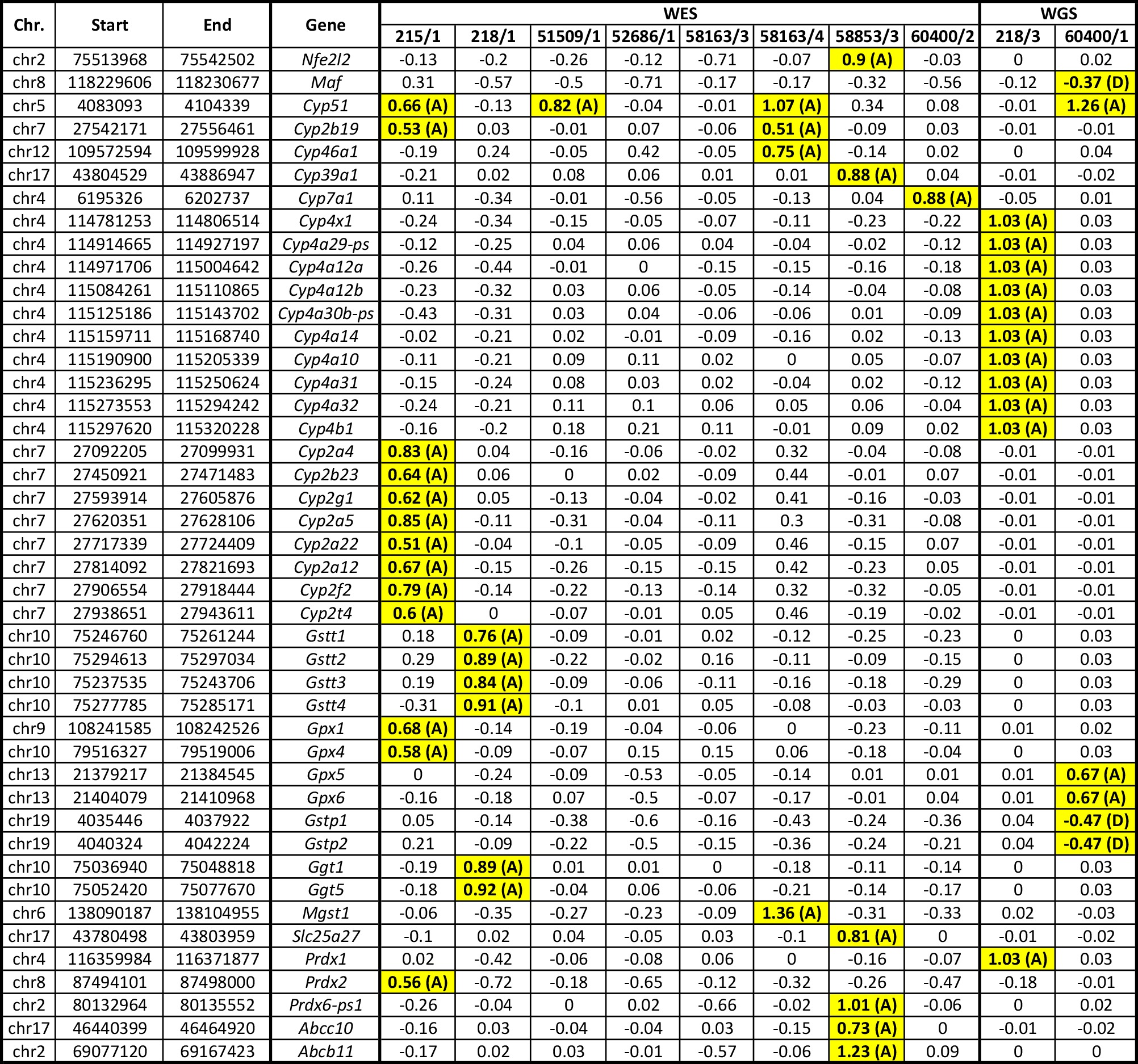

Supplement: S5 Fig — Log2 ratios between normalized gene coverage in tumoral and reference DNA in nodule samples that underwent whole exome sequencing (WES) or whole genome sequencing (WGS) are reported. Amplified or deleted regions are highlighted in yellow. Data are from Iannelli et al., 2014. (TIF) [file pgen.1007380.s005.tif]

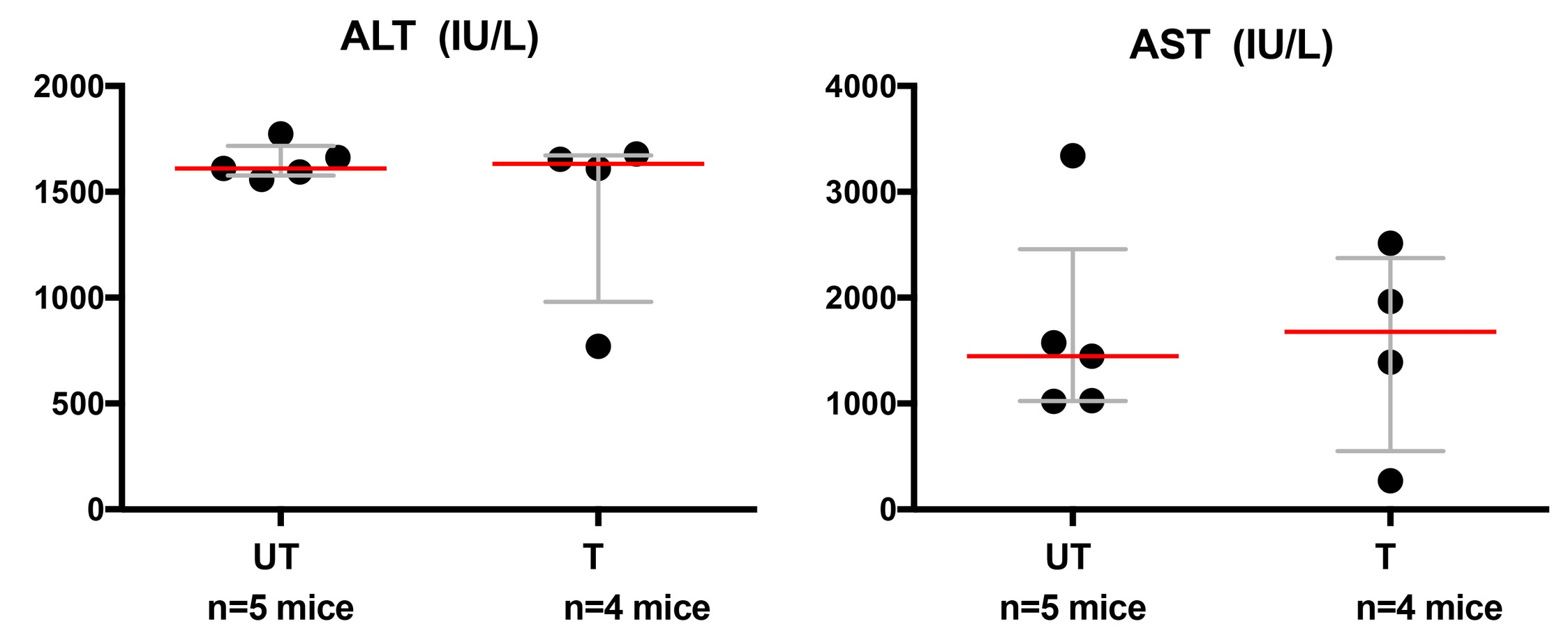

Supplement: S6 Fig — Note that one sample of treated mouse was excluded from the assay because of erythrocyte hemolysis. (TIF) [file pgen.1007380.s006.tif]

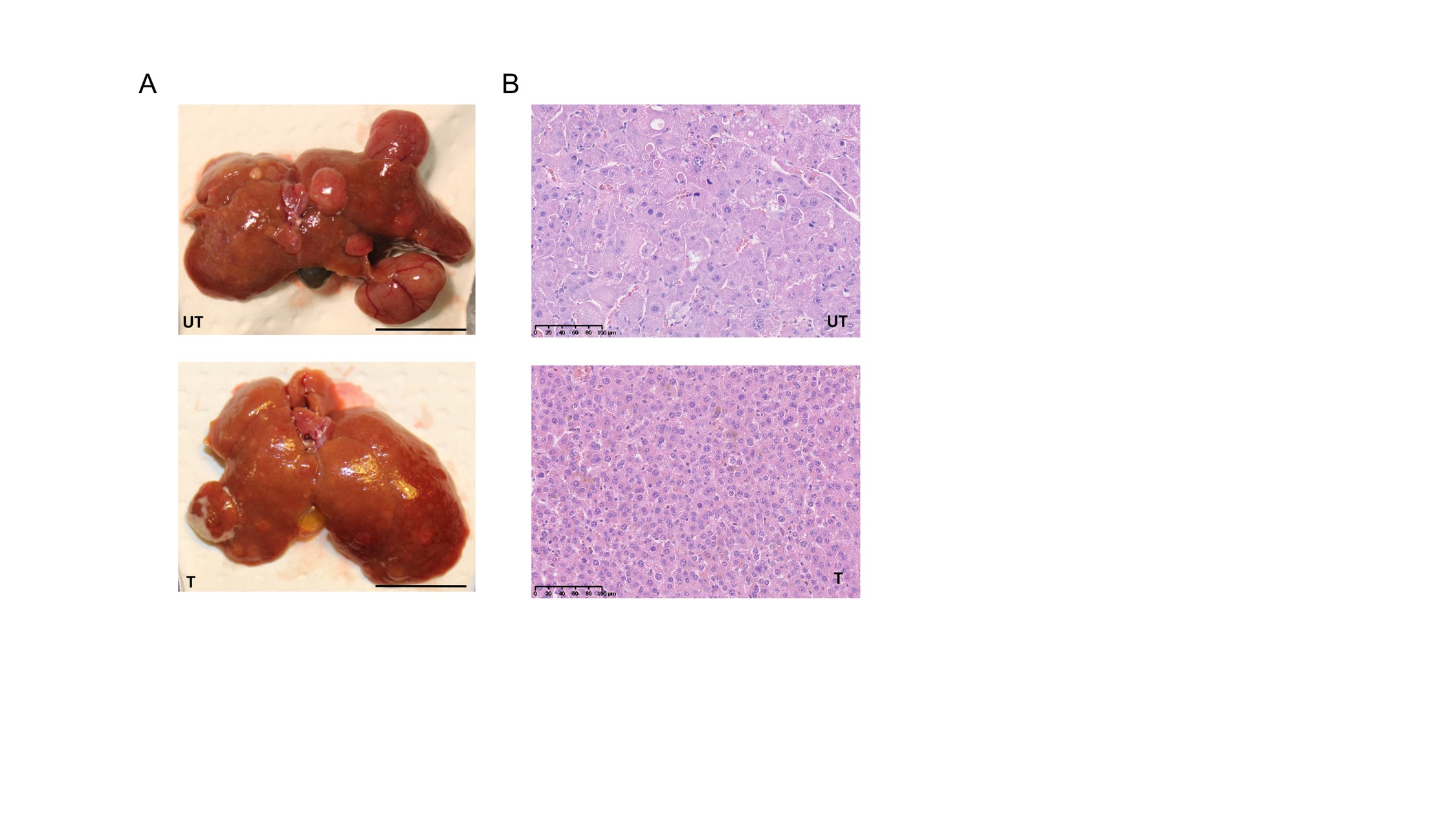

Supplement: S7 Fig — (A) Representative images of livers from an untreated (UT) and a treated (T) Mdr2-/- mouse. Scale bar = 1cm. (B) Representative hematoxylin/eosin histologic sections of HCC and adenoma from untreated and treated livers. Scale bar = 100 um. (TIF) [file pgen.1007380.s007.tif]
